# Classificação antropométrica de gestantes: comparação entre cinco métodos diagnósticos utilizados na América Latina

**DOI:** 10.26633/RPSP.2017.85

**Published:** 2017-06-19

**Authors:** Suzana Lins da Silva, Cristiane Campello Bresani-Salvi, Maria de Fátima Costa Caminha, José Natal Figueiroa, Malaquias Batista Filho

**Affiliations:** 1 Instituto de Medicina Integral Prof. Fernando Figueira (IMIP) Grupo de Pesquisa Estudos da Nutrição Recife (PE) Brasil Instituto de Medicina Integral Prof. Fernando Figueira (IMIP), Grupo de Pesquisa Estudos da Nutrição, Recife (PE), Brasil.; 2 Instituto Nacional do Seguro Social (INSS) Serviço de Saúde do Trabalhador da GEX Recife (PE) Brasil Instituto Nacional do Seguro Social (INSS), Serviço de Saúde do Trabalhador da GEX, Recife (PE), Brasil.

**Keywords:** Antropometria, diagnóstico, gestação, índice de massa corporal, sobrepeso, América Latina, Anthropometry, diagnosis, pregnancy, body mass index, overweight, Latin America, Antropometría, diagnóstico, embarazo, índice de masa corporal, sobrepeso, América Latina

## Abstract

**Objetivo.:**

*Verificar a concordância entre cinco métodos antropométricos na classificação nutricional de gestantes e comparar as classificações obtidas com a classificação nutricional da população brasileira de mulheres jovens não gestantes*.

**Método.:**

*Estudo transversal com dados de 1 108 gestantes com idade de 19 a 35 anos atendidas de setembro de 2011 a abril de 2012 em serviços de pré-natal no estado de Pernambuco, Brasil. A classificação nutricional (baixo peso, peso adequado e sobrepeso/obesidade) foi realizada conforme os critérios de Mardones e Rosso, de Mardones et al., de Atalah et al., do Centro Latino Americano de Perinatologia* (CLAP) *e do* Institute of Medicine *de 2009* (IOM-2009)*. Estimaram-se os coeficientes kappa de concordância para os pares de métodos. O teste do qui-quadrado de bondade de ajuste foi utilizado para comparar a distribuição de frequências de cada categoria nutricional em cada método com a distribuição em não gestantes classificadas de acordo com índice de massa corporal (IMC, pontos de corte da OMS)*.

**Resultados.:**

*Os métodos concordaram entre si para o diagnóstico de sobrepeso/obesidade (kappa > 0,60) e discordaram em relação ao baixo peso (kappa* ≤ *0,60), particularmente nas comparações do IOM-2009 (que utiliza o IMC pré-gestacional) com os demais. As distribuições de frequências amostrais obtidas com os cinco métodos diferiram da população de referência de não gestantes (*P *< 0,001), observando-se percentuais de sobrepeso/obesidade inferiores à prevalência nacional e percentuais de baixo peso superiores à prevalência nacional*.

**Conclusão.:**

*As disparidades observadas podem ser atribuídas à heterogeneidade entre os métodos, justificando a realização de inquéritos para definir padrões antropométricos específicos para determinadas populações*.

## Abstract (en)

***Objective*.:**

To determine the agreement between five anthropometric methods used for nutritional assessment in pregnancy and to compare the distribution of nutritional status obtained with each method to that of the population of non-pregnant young women in Brazil.

This is a cross-sectional study with data from 1 108 pregnant women aged 19 to 35 years who received prenatal care from September 2011 to April 2012 in health services in the state of Pernambuco, Brazil. Nutritional status (underweight, appropriate weight, overweight/obesity) was determined using the criteria of Mardones and Rosso, Mardones et al., Atalah et al., *Centro Latino Americano de Perinatologia* (CLAP), and the Institute of Medicine (IOM-2009). Kappa agreement was estimated for the pairs of methods, and the chi-square goodness of fit test was performed to compare the frequency distribution of each nutritional category in each of the methods in comparison to the distribution in non-pregnant women classified according to body mass index (BMI, WHO cut-off points).

Agreement between the methods was observed for overweight/obesity (kappa > 0.60), but not for underweight (kappa ≤ 0.60), particularly in the comparison of IOM-2009 (which relies on prepregnancy BMI) with other methods. The frequency distributions obtained with the five methods showed lower percentages of overweight/obesity and higher percentages of underweight as compared to the reference population of non-pregnant women (*P* < 0.001),

***Conclusion*.:**

The disparities observed in the present study may have resulted from the heterogeneity among the methods. This suggests that additional surveys are needed to establish population-specific anthropometric standards.

## RESUMEN

**Objetivo.:**

Verificar si hay concordancia entre cinco métodos antropométricos de clasificación nutricional de las embarazadas y comparar las clasificaciones obtenidas con la clasificación nutricional de la población brasileña de mujeres jóvenes no embarazadas.

**Método.:**

Estudio transversal con datos de 1 108 embarazadas de 19 a 35 años atendidas desde septiembre del 2011 hasta abril del 2012 en servicios de atención prenatal en el estado de Pernambuco (Brasil). La clasificación nutricional (peso bajo, peso adecuado y sobrepeso u obesidad) se realizó de acuerdo con los criterios de Mardones y Rosso, de Mardones et al., de Atalah et al., del Centro Latinoamericano de Perinatología (CLAP) y del Instituto de Medicina del 2009 (IOM-2009). Se estimaron los coeficientes kappa de concordancia para los pares de métodos y la prueba del ji-cuadrado de la bondad del ajuste para comparar la distribución de frecuencias de cada categoría nutricional obtenida con cada método con la distribución en las mujeres que no están embarazadas clasificadas según el índice de masa corporal (IMC, puntos de corte de la OMS).

**Resultados.:**

Los métodos concordaron en lo que respecta al diagnóstico de sobrepeso y obesidad (kappa 0,60) y no concordaron en relación con el peso bajo (kappa ≤ 0,60), particularmente cuando se compararon las clasificaciones basadas en los criterios de la IOM-2009 (que utiliza el IMC pregestacional) con los demás. Las distribuciones de frecuencias muestrales obtenidas con los cinco métodos difirieron de la población de referencia de mujeres no embarazadas (P < 0,001), observándose porcentajes de sobrepeso y obesidad inferiores a la prevalencia nacional y porcentajes de peso bajo superiores a la prevalencia nacional.

**Conclusión.:**

Las disparidades observadas pueden atribuirse a la heterogeneidad de los métodos. Se justifica la realización de investigaciones para definir patrones antropométricos específicos para determinadas poblaciones.

Embora a gestação seja um processo fisiológico, seu desenvolvimento implica em marcantes alterações hormonais, metabólicas, morfofuncionais e psicocomportamentais, configurando um ciclo de reconhecida vulnerabilidade para a mãe e para o feto, principalmente nos países em desenvolvimento (1). Em contextos de pobreza, a carga simultânea de carência nutricional e de excesso calórico podem constituir-se em risco obstétrico (2–4). No entanto, a despeito de sua variabilidade epidemiológica e de sua relação com desfechos gestacionais negativos (restrição de crescimento intrauterino, baixo peso ao nascer, macrossomia, prematuridade e mortalidade perinatal) (5, 6), o estado nutricional da gestante não tem recebido a atenção devida no campo da investigação científica e da vigilância nutricional (7).

No caso da classificação nutricional de gestantes baseada na antropometria, a diversidade de métodos limita a compilação de estimativas e o direcionamento das ações de saúde (8, 9). Na América Latina, sete instrumentos de classificação baseados em peso, estatura e idade gestacional têm sido aplicados na prática clínica do pré-natal (10–12): 1) método de Rosso (13); 2) método do Centro Latino-Americano de Perinatologia (CLAP)(14); 3) método do *Institute of Medicine* de 1992 (IOM-1992) (15); 4) nomograma de Atalah et al. (16); 5) nomograma de Mardones e Rosso (17); 6) nomograma de Mardones et al. (18); e 7) método do IOM de 2009 (19). Esses instrumentos de avaliação foram elaborados e testados em populações com características específicas, havendo escassez de estudos de validação nos países da América Latina, o que leva a questionamentos sobre a sua validade e comparabilidade ampliadas a contextos epidemiológicos com fenótipos e condições socioambientais distintos (20–22).

De fato, estudos têm relatado discordâncias na avaliação nutricional de gestantes por esses diferentes instrumentos (9). Além disso, há indícios de que a antropometria tende a superestimar as prevalências de baixo peso e subestimar sobrepeso e obesidade gestacionais (20). A propósito, um estudo realizado no Nordeste brasileiro há mais de 10 anos (23) destacou discrepâncias entre as frequências de baixo peso, sobrepeso e obesidade ao comparar gestantes classificadas de acordo com os critérios de Rosso (13), Atalah et al. (16) ou CLAP (14) com não gestantes classificadas pelo critério de índice de massa corporal (IMC) da Organização Mundial da Saúde (OMS).

Tendo em vista a carência de estimativas de validação das classificações de maior uso na atualidade no nosso continente, o objetivo do presente estudo foi comparar a classificação nutricional de gestantes obtida com esses métodos antropométricos. Além disso, partindo-se do pressuposto de que a gestação fisiológica não deveria modificar a antropometria a ponto de alterar a classificação nutricional pré-gestacional da mulher, realizamos uma comparação entre a classificação nutricional obtida para as gestantes com cada método e a classificação nutricional de jovens brasileiras não gestantes.

## MATERIAIS E MÉTODOS

Trata-se de um estudo transversal que comparou a classificação nutricional de gestantes atendidas em três serviços de pré-natal obtida com cinco métodos antropométricos utilizados na América Latina: nomogramas de Atalah et al. (16), Mardones e Rosso (17) e Mardones et al. (18), IOM-2009 (19) e CLAP (14). Apesar de o método do CLAP ter sido construído com medidas seriadas de apenas 43 gestantes e de seu progressivo desuso, optamos por incluí-lo, por ter sido o mais compatível com a classificação de não gestantes em um estudo anterior na nossa região (23). A classificação das gestantes obtida com cada um desses métodos foi ainda comparada com a classificação da população brasileira de mulheres jovens (idade de 19 a 35 anos) não gestantes obtida pelo critério da OMS: baixo peso, IMC < 18,5 kg/m^2^; peso adequado, IMC de 18,5 a 24,9 kg/m^2^; sobrepeso, IMC de 25,0 a 29,9 kg/m^2^; e obesidade, IMC≥ 30,0 kg/m^2^ (24).

Este estudo utilizou o banco de dados primários do inquérito “Estado nutricional de gestantes: aspectos metodológicos, epidemiológicos e implicações na assistência pré-natal”, realizado pelo Grupo de Pesquisa Estudos da Nutrição do Instituto de Medicina Integral Prof. Fernando Figueira (IMIP) e pelo Departamento de Nutrição da Universidade Federal de Pernambuco (UFPE) (25). O projeto original teve como objetivo descrever a situação nutricional (anemia, deficiência de vitamina A e classificação antropométrica) de gestantes atendidas em três serviços de assistência pré-natal do estado de Pernambuco: Centro de Assistência à Mulher do IMIP, localizado em Recife, capital do estado, e unidades de assistência pré-natal de Vitória de Santo Antão (zona da Mata) e de Caruaru (Agreste Pernambucano). A coleta de dados foi realizada de setembro de 2011 a abril de 2012.

A população alvo incluiu mulheres em acompanhamento pré-natal de baixo risco, provenientes da região metropolitana e da zona rural do estado de Pernambuco. Na III Pesquisa Estadual de Saúde e Nutrição (III PESN 2006) (26), a média de idade da população de mulheres não gestantes adultas jovens (19 a 35 anos) de Pernambuco foi de 27 anos, sendo que 75% tinham até 9 anos de escolaridade, 55% possuíam renda *per capita* abaixo de um quarto de salário mínimo, 30% eram primíparas e 54% apresentavam IMC adequado.

A amostra final do estudo primário foi calculada com base no desenho transversal descritivo para detectar prevalências de 40% de anemia (erro amostral ± 3%) em gestantes brasileiras (27), 15% de deficiência de vitamina A (erro amostral de ± 2%) (28) e 45% de sobrepeso/obesidade (erro amostral ± 3%) na população de gestantes pernambucanas (26). Uma amostra de 1 200 gestantes seria necessária para estimar a menor dessas proporções (15% de deficiência de vitamina A) com nível de significância de 95% (1-α). Ao final do inquérito, haviam sido recrutadas 1 516 gestantes, considerando-se uma perda de dados em torno de 20%.

Para o presente estudo, selecionaramse, a partir do banco de dados original, todas as gestantes que atendiam os critérios de inclusão (idade de 19 a 35 anos e ter registro sobre peso e estatura), excluindo-se aquelas de alto risco, o que resultou em uma amostra de 1 108 participantes. A faixa etária adotada para essas análises objetivou excluir o período da adolescência, caracterizado por particularidades antropométricas, e a idade materna acima de 35 anos, que implica gestações de alto risco.

## Métodos de classificação antropométrica

O estado nutricional das gestantes foi classificado nas categorias de baixo peso, peso adequado e sobrepeso/obesidade segundo cada um dos métodos de estudo. A classificação pelo método de Mardones e Rosso (17) baseia-se no percentual do peso padronizado (= peso observado x 100/peso esperado para altura) por semana gestacional de acordo com um nomograma, iniciando com pontos de corte para a 10^a^ semana gestacional: baixo peso, < 95% do previsto no nomograma; peso adequado, 95 a 109%; sobrepeso, 110 a 119%; e obesidade, ≥ 120%. Na 40^a^ semana, a classificação prevê como baixo um peso < 119,2% do nomograma, peso adequado de 119,2 a 129,7%, sobrepeso de 129,8 a 134,7% e obesidade > 134,7% do previsto no nomograma.

A classificação pelo método de Mardones et al. (18) aplica o IMC por semana gestacional em um nomograma que inicia na 10^a^ semana gestacional (baixo peso, < 21,1 kg/m^2^; peso adequado, 21,1 a 24,4 kg/m^2^; sobrepeso 24,5 a 26,7 kg/m^2^; e obesidade ≥ 26,7 kg/m^2^) e termina na 40^a^ semana (baixo peso, < 26,5 kg/m^2^; peso adequado, 26,5 a 28,9 kg/m^2^; sobrepeso, 29,0 a 30,0 kg/m^2^; e obesidade, ≥ 30,0kg/m^2^).

O método de Atalah et al. (16) também utiliza um nomograma, aplicando pontos de corte do IMC por semana gestacional para classificar a mulher a partir da 6^a^ semana de gestação (baixo peso, < 19,9 kg/m^2^; peso adequado, 20,0 a 24,9 kg/m^2^; sobrepeso, 25,0 a 30,0 kg/m^2^; e obesidade, ≥ 30,1 kg/m^2^) até a 42^a^ semana (baixo peso, < 25,0 kg/m^2^; peso adequado, 25,1 a 29,2 kg/m^2^; sobrepeso, 29,3 a 33,2 kg/m^2^; e obesidade, ≥ 33,3kg/m^2^).

O método do CLAP (14) relaciona intervalos de peso com intervalos de estatura em cada semana gestacional desde a 13^a^ até a 39^a^ semana, classificando a gestante de acordo com os seguintes percentis: baixo peso < P10, peso adequado P10 a P90 e sobrepeso/obesidade > P90.

O método do IOM-2009 (19) propõe classificar o estado de nutrição antes da gestação com base no IMC pré-gestacional para então prescrever o ganho de peso adequado semanal e total para cada categoria: baixo peso, peso adequado, sobrepeso e obesidade. Para classificar o estado nutricional pré-gestacional, o IOM-2009 utiliza o IMC calculado com peso e estatura informados ou aferidos no 1° trimestre de gestação e classifica as gestantes em baixo peso (IMC < 18,5 kg/m^2^), peso adequado (18,5 ≤ IMC ≤ 24,9 kg/m^2^), sobrepeso (25,0 ≤ IMC ≤ 29,9 kg/m^2^) e obesidade (IMC ≥ 30 kg/m^2^). Assim, o método recomenda um ganho de peso total adequado de 12,5 kg a 18 kg para as gestantes classificadas como baixo peso; de 11,5 kg a 16,0 kg para as classificadas como peso adequado; de 7,0 a 11,5 kg nas classificadas com sobrepeso; e de 5,0 a 9,0 kg nas obesas.

No nosso estudo, a classificação das gestantes pelos métodos Mardones e Rosso (17), Mardones et al. (18), CLAP (14) e Atalah et al. (16) utilizou peso e estatura aferidos, enquanto que a classificação pelo IOM-2009 (19) utilizou peso e estatura informados. Essa conduta foi corroborada por uma análise de concordância realizada na subamostra de 159 gestantes que estavam no 1^o^ trimestre de gestação. Para essa subamostra havia registros de peso e altura informados e aferidos. Os coeficientes de correlação intraclasse foram iguais a 0,94 para o peso e 0,97 para estatura. Esses valores indicaram uma concordância muito boa entre peso informado e peso aferido, bem como entre estatura informada e aferida.

O presente estudo partiu do pressuposto de que o estado nutricional na gravidez, com suas variações para mais ou para menos, deve refletir a condição prégestacional, que, por sua vez, deveria coincidir com a distribuição de frequências esperada para mulheres brasileiras não gestantes em idade adulta jovem. Assim, com base na convenção bioestatística de normalidade, a classificação nutricional conforme peso e estatura prégestacionais da amostra (método do IOM-2009) (19), assim como a classificação da população brasileira de não gestantes, foram consideradas como os padrões de referência nas análises e interpretações.

## Dados e análises

Um questionário pré-testado foi aplicado por entrevistadores treinados para coletar dados sobre características sociodemográficas (idade, anos de estudo, renda mensal *per capita*) e obstétricas (número de gestações anteriores, data da última menstruação e número de consultas de pré-natal) e medidas antropométricas (peso e estatura). A renda mensal *per capita* foi calculada em salários mínimos. No período entre 2011 e 2012, o valor do salário mínimo em reais (moeda brasileira) era de R$ 622,00, ou US$ 342,69, de acordo com o câmbio monetário no mesmo período (US$ 1,00 = R$ 1,815) (29).

A idade gestacional foi calculada utilizando a estimativa do exame ultrassonográfico no 1° trimestre de gravidez, e, na ausência desse exame, a data da última menstruação. Para aferição dos dados antropométricos (peso e estatura das gestantes), usou-se balança digital da marca Plenna®, com leitura para 100 gramas. A estatura foi medida em estadiômetro (Alturaexata) com divisões em centímetros e milímetros e leitura aproximada para valores inteiros ou fracionários (0 ou 0,5 cm). As gestantes foram medidas e pesadas descalças, sem objetos nas mãos ou nos bolsos (30). Antes da realização das medidas antropométricas, as gestantes informavam seu peso e estatura antes de engravidarem.

As análises estatísticas foram realizadas com o programa Stata versão 12.1SE. Em todos os testes foi adotado um nível de significância de 0,05. Foram calculadas as frequências absolutas e relativas da classificação antropométrica com cada método. As concordâncias entre pares de métodos de classificação gestacional foram avaliadas pelo coeficiente kappa com intervalo de confiança de 95% (IC95%) com base na escala proposta por Landis e Koch (31): concordância fraca (0,00 a 0,20), regular (0,21 a 0,40), moderada (0,41 a 0,60), boa (0,61 a 0,80) e muito boa (0,81 a 1,00). Nas análises de concordância do método do CLAP com os demais métodos, foram excluídas as observações das mulheres com estatura fora do intervalo de 140 a 169 cm ou idade gestacional abaixo de 13 ou acima de 39 semanas, devido aos limites dos critérios do CLAP (14). Além disso, o sobrepeso e a obesidade foram agrupados em uma única categoria (sobrepeso/obesidade), já que esse método não distingue as duas situações.

Por fim, as frequências obtidas com a classificação das gestantes utilizando-se cada um dos métodos foram comparadas com a classificação de não gestantes brasileiras da Pesquisa Nacional de Saúde do IBGE/Ministério da Saúde de 2013 (32). Os dados referentes a mulheres jovens não gestantes disponíveis no relatório do IBGE estão agregados em um intervalo de idade de 18 a 34 anos, aproximando-se da faixa etária na nossa amostra de gestantes (19 a 35 anos). Cabe observar que, em anos recentes, as diferenças regionais dos padrões antropométricos de mulheres brasileiras em idade reprodutiva foram significativamente reduzidas, evoluindo, de fato, para uma homogeneização em escala nacional (33).

Utilizamos o teste do qui-quadrado (χ^2^) de bondade de ajuste com a finalidade de comparar a classificação nutricional das gestantes (frequências amostrais) com a das não gestantes (probabilidades esperadas) (32). Nesse teste, um valor de *P* < 0,05 implica que, em nível de significância de 5%, a distribuição amostral não é compatível com a distribuição esperada. Obtivemos como distribuição esperada na população de não gestantes as frequências de 5,5% para baixo peso, 33,5% para peso adequado, 44,3% para sobrepeso e 16,7% para obesidade, segundo a classificação do IMC pelo critério da OMS (24).

Como as classificações foram realizadas *a posteriori* em banco de dados, não foi possível tomar condutas quanto aos desvios nutricionais, nem tampouco identificar as puérperas e comunicar-lhes os resultados. O estudo foi aprovado pelo Comitê de Ética em Pesquisa com Seres Humanos do IMIP (n° CAAE: 13448413.6.0000.5201). Todas as participantes assinaram um termo de consentimento livre e esclarecido. A confidencialidade e o anonimato foram garantidos através do manuseio do banco de dados em local sigiloso, exclusivamente pelos autores, sendo as participantes registradas com códigos únicos.

## RESULTADOS

A tabela 1 descreve as características sociodemográficas, obstétricas e as medidas antropométricas da população amostral de estudo, reunindo um mínimo de 1 068 observações em relação à renda *per capita* e um máximo de 1 108 referentes ao registro de idade, trimestre de gravidez e estatura das gestantes. Para as classificações nutricionais, foram incluídas 1 108 gestantes no método de Atalah et al., 1 071 gestantes no método de Mardones e Rosso e Mardones et al., 1 070 no método do IOM e 877 no método do CLAP.

**TABELA 1.** Características sociodemográficas e obstétricas e medidas antropométricas de gestantes atendidas em serviços de pré-natal no estado de Pernambuco, Brasil, 2011 e 2012

Variável	No.	%
Idade (anos)	(n = 1 108)	
19 a 24	525	47,4
25 a 29	326	29,4
30 a 35	257	23,2
Escolaridade (anos)	(n = 1 098)	
Até 9 anos	294	26,8
10 ou mais	804	73,2
Renda mensal *per capita* (salários mínimos)^a^	(n = 1 068)	
< 0,25	196	18,4
0,25 a 0,50	442	41,4
> 0,50	430	40,2
Número de gestações	(n = 1 107)	
1	490	44,3
2 ou 3	522	47,1
4 ou mais	95	8,6
Trimestre gestacional	(n = 1 108)	
Primeiro	159	14,4
Segundo	666	60,1
Terceiro	283	25,5
Consultas no pré-natal	(n = 1 105)	
1 a 3	794	71,9
4 a 5	248	22,4
≥ 6	63	5,7
Classificação nutricional pré-gestacional^b^	(n = 1 070)	
Baixo peso	90	8,4
Adequado	613	57,3
Sobrepeso	254	23,7
Obesidade	113	10,6
Estatura (cm)	(n = 1 108)	
< 150	56	5,0
150 a 160	577	52,1
≥ 160	475	42,9

a Salário mínimo correspondente a US$ 342,70 no momento da entrada no estudo.

b Índice de massa corporal (IMC) pré-gestacional de acordo com o método do *Institute of Medicine* (IOM-2009) (19).

Na tabela 2 observa-se que o maior coeficiente de correlação kappa ocorreu para a comparação entre as duas versões de Rosso e Mardones, atingindo concordância muito boa (kappa > 0,80) tanto para baixo peso como para sobrepeso/obesidade. Para os demais pares de comparações, as concordâncias foram regulares ou moderadas (kappa ≤ 0,60) para o diagnóstico de baixo peso e boas ou muito boas (kappa > 0,60) para o sobrepeso/obesidade. As frequências de baixo peso, peso adequado, sobrepeso e obesidade diferiram entre os métodos de classificação, exceto entre as duas versões de Rosso e Mardones, sendo que ambas indicaram que um quarto das mulheres teria baixo peso e cerca de 50% teriam sobrepeso/obesidade (tabela 3).

**TABELA 2.** Análise de concordância (kappa) entre cinco classificações antropométricas gestacionais aplicadas a gestantes atendidas em serviços de pré-natal, estado de Pernambuco, Brasil, 2011 e 2012

Classificação^a^	No. de gestantes na análise	Total	Baixo peso	Sobrepeso/obesidade
		kappa (IC95%)^b^	kappa (IC95%)^b^	kappa (IC95%)^b^
IOM-2009 vs. Mardones e Rosso	1 070	0,46 (0,42 a 0,49)	0,37 (0,31 a 0,44)	0,59 (0,55 a 0,64)
IOM-2009 vs. Mardones et al.	1 070	0,46 (0,42 a 0,50)	0,35 (0,28 a 0,41)	0,62 (0,57 a 0,66)
IOM-2009 vs. Atalah et al.	1 070	0,56 (0,52 a 0,61)	0,48 (0,40 a 0,55)	0,66 (0,61 a 0,70)
IOM-2009 vs. CLAP	877	0,55 (0,50 a 0,60)	0,44 (0,34 a 0,54)	0,52 (0,47 a 0,57)
Atalah et al. vs. Mardones e Rosso	1 071	0,55 (0,51 a 0,58)	0,77 (0,72 a 0,82)	0,82 (0,79 a 0,86)
Atalah et al. vs. Mardones et al.	1 071	0,72 (0,68 a 0,76)	0,72 (0,67 a 0,77)	0,88 (0,85 a 0,90)
Atalah vs. CLAP	877	0,72 (0,69 a 0,77)	0,68 (0,60 a 0,75)	0,81 (0,77 a 0,85)
Mardones e Rosso vs. Mardones et al.	1 071	0,89 (0,86 a 0,92)	0,93 (0,90 a 0,96)	0,94 (0,92 a 0,96)
Mardones e Rosso vs. CLAP	877	0,58 (0,54 a 0,62)	0,49 (0,42 a 0,56)	0,94 (0,92 a 0,96)
Mardones et al. vs. CLAP	877	0,60 (0,63 a 0,71)	0,45 (0,38 a 0,52)	0,92 (0,90 a 0,94)

a IOM-2009: *Institute of Medicine* 2009 (19); CLAP: Centro Latino Americano de Perinatologia (14); Atalah et al. (16); Mardones e Rosso (17); Mardones et al. (18).

b Valor *P* para o teste kappa de concordância: < 0.001 para todos os testes. Concordância conforme escala proposta por Landis e Koch (31): fraca (0,00 a 0,20), regular (0,21 a 0,40), moderada (0,41 a 0,60), boa (0,61 a 0,80) e muito boa (0,81 a 1,00).

**TABELA 3:** Classificação antropométrica de gestantes a partir de cinco métodos comparada com classificação de brasileiras jovens, não gestantes, de acordo com o índice de massa corporal pelo critério da Organização Mundial da Saúde, Pernambuco, Brasil, 2011 e 2012

Categoria de peso	Método de classificação antropométrica de gestantes^a^					
	Não gestantes^b^	IOM-2009 No. = 1 070	CLAP No. = 877	Atalah et al. No. = 1 108	Mardones e Rosso No. = 1 071	Mardones et al. No. = 1 071
Baixo peso	5,5	8,4	9,0	15,9	23,2	25,2
Adequado	33,5	57,1	39,9	42,1	27,3	27,8
Sobrepeso/obesidade	61,0	34,3	51,1	42,0	49,6	47,0
*P* ^c^		< 0,001	< 0,001	< 0,001	< 0,001	< 0,001	

a IOM-2009: Institute of Medicine 2009 (19); CLAP: Centro Latino Americano de Perinatologia (14); Atalah et al. (16); Mardones e Rosso (17); Mardones et al. (18).

b Não gestantes classificadas de acordo com o índice de massa corporal pelo critério da Organização Mundial da Saúde (24). Pontos de corte: baixo peso (IMC < 18,5 kg/m^2^), peso adequado (18,5 ≤ IMC ≤ 24,9 kg/m^2^), sobrepeso (25,0 ≤ IMC ≤ 29,9 kg/m^2^) e obesidade (IMC ≥ 30 kg/m^2^).

c Teste do qui-quadrado de bondade de ajuste para a comparação entre frequências observadas e frequência esperada conforme Pesquisa Nacional de Saúde (32).

**FIGURA 1.**
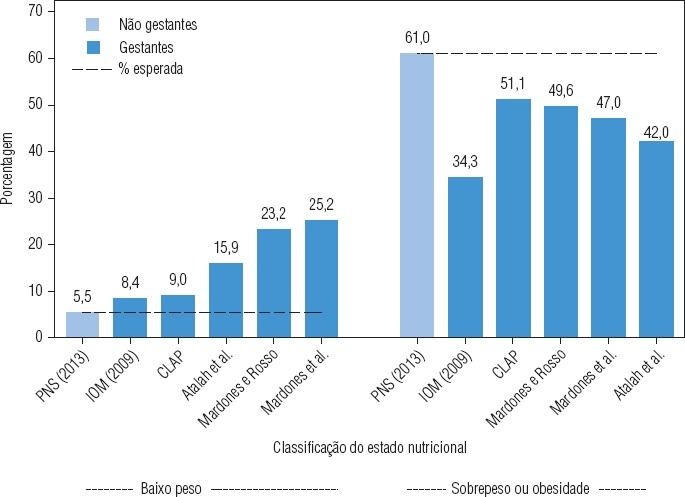
Frequência de baixo peso e sobrepeso/obesidade em não gestantes e em gestantes classificadas de acordo com cinco métodos de avaliação antropométrica, Brasil, 2011 e 2012^a^.

^a^**PNS**: Pesquisa Nacional de Saúde (32); IOM: *Institute of Medicine* (19); CLAP: Centro Latino Americano de Perinatologia (14); Atalah et al. (16); Mardones e Rosso (17); Mardones et al. (18). Método do IOM-2009 baseado no índice de massa corporal pré-gestacional. CLAP relaciona intervalos de peso com intervalos de estatura em cada semana gestacional. Método de Mardones e Rosso baseia-se no percentual do peso padronizado (= peso observado x 100/peso esperado para altura). Mardones et al. e Atalah et al. baseados no índice de massa corporal por idade gestacional.

Os percentuais das categorias nutricionais obtidas com todos os métodos diferiram significativamente das frequências esperadas com base no IMC da população de não gestantes (valor de *P* do teste do χ^2^ de bondade de ajuste < 0,001). A figura 1 ilustra visualmente essas diferenças, mais acentuadas para o baixo peso, cujas razões de prevalências chegaram a variar de quase duas a cinco vezes em relação às classificações do CLAP (9,0 *versus* 5,5%), de Atalah (15,9% *versus* 5,5%), Mardones e Rosso (23,2% *versus* 5,5%) e Mardones et al. (25,2% *versus* 5,5%).

## DISCUSSÃO

Nas gestantes estudadas, os métodos de classificação antropométrica, no geral, concordaram entre si para o diagnóstico de sobrepeso/obesidade e discordaram em relação ao baixo peso, particularmente nas comparações do IMC pré-gestacional (método do IOM-2009) (19) com os demais. As distribuições de frequências amostrais de baixo peso, peso adequado e sobrepeso/obesidade obtidas com todos os métodos não foram compatíveis com os resultados da população de referência de não gestantes. Os métodos baseados em peso-estatura gestacional geraram frequências de sobrepeso/obesidade inferiores à prevalência na população de referência, enquanto o baixo peso apresentou ampla variação, com percentuais superiores à estimativa nacional (34). O IMC pré-gestacional (IOM-2009) resultou nas mais baixas frequências das duas alterações nutricionais, aproximando-se da prevalência nacional para baixo peso e afastando-se da prevalência nacional de sobrepeso/obesidade (metade do esperado) (34).

Parte dessas discordâncias pode ter sido originada nas diferenças metodológicas entre os cinco modelos de classificação (35, 36). A esse respeito, a metodologia de Rosso e Mardones, Mardones et al. (17, 18) e Atalah et al. (16) baseou-se em gestantes chilenas, a do CLAP baseou-se em gestantes uruguaias (14) e a classificação do IOM-2009 (19) foi derivada de populações de países desenvolvidos. Com relação aos parâmetros, a classificação de Rosso e Mardones (17) adota o percentual peso/estatura, enquanto que a de Mardones et al. (18) e a de Atalah et al. (16) utilizam o IMC, porém com pontos de corte distintos. Como exemplo, na 10^a^ semana de gestação, casos fora da faixa de peso adequado (IMC de 21,1 a 24,4 kg/m^2^) por Mardones et al. (18) estariam na faixa adequada (IMC de 20,3 a 25,2 kg/m^2^) de acordo com Atalah et al. (16). Por sua vez, os pontos de corte desses dois métodos para baixo peso e sobrepeso/obesidade durante a gravidez situam-se acima dos pontos de corte da OMS (IMC baixo peso < 18,5 kg/m^2^; sobrepeso/obesidade > 24,9 kg/m^2^) (24), o que os torna mais sensíveis do que o método do IOM-2009 para o diagnóstico do déficit nutricional e menos sensíveis para o sobrepeso/obesidade (19).

A falta de um padrão ouro para definir excesso e déficit nutricionais na gestação dificulta a realização de estudos de acurácia diagnóstica dos instrumentos baseados na relação peso-estatura. Sendo assim, análises comparativas entre os métodos existentes, como as apresentadas neste estudo e em estudos prévios, são úteis para verificar precisão e confiabilidade. Um estudo conduzido no Nordeste do Brasil relatou frequências de baixo peso distintas e acima da prevalência nacional ao classificar gestantes com os métodos de Rosso (40%), de Atalah et al. (18%) e do CLAP (20%) (23), em conformidade com nossos resultados. Outro estudo relatou que o IMC pré-gravídico pelo método do IOM superestimou o sobrepeso em gestantes adolescentes do Sudeste do Brasil (37) em comparação com o critério dos *Centers for Disease Control and Prevention 2000* (38). Alguns autores têm avaliado a acurácia desses instrumentos para predizer o baixo peso ao nascer (21, 22, 39). Kac et al. (39) observaram baixo poder discriminatório da classificação de Atalah et al. (área sob a curva ROC < 0,7) (16), enquanto Barros et al. (21) e Padilha et al. (22) encontraram sensibilidade e especificidade em torno de 70% pelo método do IOM.

Com base no pressuposto de que o quadro epidemiológico antropométrico de não gestantes deveria refletir-se nas estimativas gestacionais de uma mesma população, a classificação do IOM-2009 (IMC pré-gestacional) pareceu mais apropriada para detectar o baixo peso gestacional em nossa realidade, enquanto os métodos baseados em peso-estatura gestacionais foram mais adequados para classificar o excesso de peso, já que concordaram entre si e apresentaram estimativas próximas à esperada. Por sua vez, os altos percentuais de baixo peso encontrados com os métodos de Rosso e Mardones (17), Mardones et al. (18) e de Atalah et al. (16) indicam baixa precisão e pouca confiabilidade para detectar déficit ponderal. Nesse contexto, é importante observar o fenômeno da transição nutricional que vem ocorrendo tanto nos países desenvolvidos quanto nos países em desenvolvimento. Dentre esses, o Brasil foi marcado, nos últimos 40 anos, pela redução do baixo peso feminino, que atingiu prevalências consideradas aceitáveis (< 5%), e pela duplicação da prevalência de excesso de peso (40–44), havendo indícios de aumento também entre gestantes (5, 45). Além das limitações específicas de cada instrumento, um problema comum a todos é a ausência de informações sobre a composição corporal (46); consequentemente, nenhuma das classificações traduz o balanço entre os ganhos de massa magra e gorda durante a gestação.

Limitações do presente estudo também podem estar implicadas nas divergências comparativas encontradas. Particularmente, nos casos de resultados relacionados com o IMC pré-gestacional (35), o peso e a estatura autorreferidos podem ter sido fonte de viés de informação. As mulheres tendem a subestimar o seu peso real (19), o que poderia explicar a reduzida frequência de sobrepeso/obesidade entre as participantes com o método do IOM-2009. Todavia, a análise de correlação dos valores informados com os aferidos para peso e estatura nas gestantes de 1º trimestre resultou em altos coeficientes, o que sugere confiabilidade interna desses dados. Quanto à validade externa, a estimativa do baixo peso pelo método do IOM-2009 pode ser corroborada pelo percentual igualmente baixo de baixa estatura amostral. Outro ponto importante relaciona-se à população adotada como referência nas comparações das distribuições de frequências. Devido à inexistência de inquéritos locais e regionais recentes, tivemos como única opção utilizar dados nacionais.

Em suma, nossas análises sugerem que o método do IOM-2009 deixou de detectar uma parcela de casos de excesso de peso gestacional e que os métodos de Rosso e Mardones, Mardones et al., Atalah et al. e CLAP classificaram parte das gestantes normais como de baixo peso. É fundamental que sejam desenvolvidos critérios antropométricos específicos para cada população, e também que sejam validados para o diagnóstico nutricional de gestantes. Para tal, recomendamos que os inquéritos de saúde e nutrição nas diferentes regiões do mundo passem a incluir amostras representativas de gestantes e que a iniciativa pública e privada fomente pesquisas sobre novos métodos de diagnóstico nutricional.

## Agradecimentos

Os autores agradecem ao Conselho Nacional de Desenvolvimento Científico e Tecnológico (CNPq) pelo apoio financeiro ao inquérito intitulado “Estado nutricional de gestantes: aspectos metodológicos, epidemiológicos e implicações na assistência pré-natal” (n° de aprovação 475868/08, em nome de MBF), que resultou no banco de dados analisado neste artigo. O CNPq não interferiu no desenho do estudo, na coleta e análise dos dados, na decisão de publicar ou na preparação do manuscrito.

## Declaração de responsabilidade

A responsabilidade pelas opiniões expressas neste manuscrito é estritamente dos autores e não reflete necessariamente as opiniões ou políticas da *RPSP/PAJPH* nem da OPAS.
